# Inpatient Outcomes of Tricuspid Transcatheter Edge-to-Edge Repair in the United States Based on Sex

**DOI:** 10.1016/j.jscai.2025.102644

**Published:** 2025-05-01

**Authors:** Amanda Nguyen, Muhammad Zia Khan, Waleed Alruwaili, Sameh Nassar, Zahoor Khan, Price Thomas, Sherif Elhosseiny, Juan Siordia, Richard Kovach, Muhammad Raza

**Affiliations:** a Department of Medicine, University of California Davis Medical Center, Sacramento, California; b Division of Cardiology, Deborah Heart and Lung Center, Browns Mills, New Jersey; c Division of Cardiology, West Virginia University Heart and Vascular Institute, Morgantown, West Virginia; d Department of Cardiology, HCA Houston Healthcare, Houston, Texas

**Keywords:** complications, outcomes, sex, tricuspid clip, tricuspid transcatheter edge-to-edge repair

## Abstract

**Background:**

Tricuspid transcatheter edge-to-edge repair (T-TEER) has emerged as an effective and safe option for the repair of tricuspid regurgitation in select patients. Prior studies on invasive and percutaneous cardiac interventions have shown differential outcomes based on sex, but specific studies investigating T-TEER outcomes on a national level are limited.

**Methods:**

The National Inpatient Sample and International Classification of Diseases, Tenth Revision codes were used to identify patients who underwent T-TEER in the US from 2018 to 2021. The study group was then stratified based on sex. Study end points assessed included inpatient complications, outcomes, and resource utilization after T-TEER. A multivariable logistic regression model was used to assess the independent association of sex with study outcomes.

**Results:**

A total of 1960 T-TEER procedures were identified, of which 1210 occurred in female patients (61.7%). Female patients were older and generally had a lower prevalence of important comorbidities than male patients. In unadjusted analysis, female sex was associated with lower prevalence of major, overall, and cardiovascular complications, inpatient mortality, and length and cost of stay. After multivariable adjustment, female sex was associated with lower inpatient mortality (adjusted odds ratio [aOR], 0.43; 95% CI, 0.22-0.82), lower major complications, (aOR, 0.69; 95% CI, 0.49-0.98), and lower cost of stay (aOR, 0.67; 95% CI, 0.55-0.82).

**Conclusions:**

Female sex was associated with similar or better inpatient outcomes and mortality after T-TEER when compared with male sex. Further investigation to understand the etiology behind these important differences is encouraged to promote improved cardiovascular care and outcomes in patients regardless of sex.

## Introduction

Tricuspid regurgitation (TR) is associated with poor cardiovascular morbidity and mortality.^1^, ^2^, ^3^ Medical treatment of TR is largely limited to diuretic use, and invasive intervention for TR is often deferred due to significantly high surgical risk.^4^^,^^5^ Tricuspid transcatheter edge-to-edge repair (T-TEER) is an innovative repair technique that has emerged as a minimally invasive option for TR repair in patients who are poor surgical candidates. Various recent studies have shown the safety and efficacy of T-TEER when compared to surgery or optimal medical therapy.^1^^,^^3^^,^^4^^,^^6^^,^^7^ Consequently, on April 2, 2024, the US Food and Drug Administration approved the TriClip (Abbott) as the first TEER system specifically designed to treat TR. Prior studies for invasive and percutaneous cardiac interventions for valvular disorders have demonstrated disparities based on sex, with female patients generally being referred to intervention later, and having worse procedural outcomes than male patients.^2^^,^^5^^,^^8^, ^9^, ^10^, ^11^ However, large, national studies investigating T-TEER outcomes stratified by sex are limited. Studies investigating these outcomes are valuable, as epidemiological studies have established that the prevalence and incidence of TR are higher in female patients than in male patients.^2^^,^^5^^,^^8^ In this study, we aimed to address this gap in the literature by investigating differences in outcomes and complications related to T-TEER among male patients and female patients in the US.

## Methods

### Data source

Data from the National Inpatient Sample (NIS) were used for the purpose of our current study. We analyzed the NIS database from years 2018 to 2021 for T-TEER procedures. The NIS is a large hospital-based administrative database that samples inpatient data from 20% of participating hospitals across the nation and is able to estimate >97% of all US hospitalizations after applying discharge weights.^12^ The NIS can be used for computing national estimates of health care utilization, costs, trends, and outcomes. The database was made possible by a Federal-State-Industry partnership sponsored by the Agency for Healthcare Research and Quality.^12^ As the data from the NIS is deidentified and publicly available, the need for informed consent and institutional review board approval is waived. The NIS adheres to the 2013 Declaration of Helsinki for the conduct of human research.

### Study population

Tricuspid transcatheter edge-to-edge repair procedures were identified using the International Classification of Diseases, 10th Revision, Clinical Modification code of 02UJ3JZ (Supplemental Appendix). Patients younger than 18 years and those with missing demographic data were excluded. The study sample was stratified on the basis of sex into male and female patients. Sex was defined as the biological sex assigned at birth, as characterized by the NIS database.

### Outcomes

Baseline characteristics, procedural complications, and inpatient outcomes including mortality (reported as a distinct categorical variable), length of stay, and hospitalization costs were compared in T-TEER procedures, stratified based on sex. We also analyzed overall complications, major complications (defined as composite of cardiovascular, vascular, hematological, and neurological complications), cardiovascular complications (defined as composite of pericardial effusion of any etiology requiring intervention, cardiac arrest, cardiogenic shock, NSTEMI/STEMI, and heart block), vascular complications (defined as composite of pseudoaneurysm, access site hematoma, retroperitoneal bleeding, and venous thromboembolism), pulmonary complications (defined as composite of acute respiratory failure, pneumothorax, and prolonged invasive ventilation), hematological complications (defined as the composite of any intraprocedural bleeding, gastrointestinal bleeding, and bleeding requiring transfusions), neurological complications (defined as the composite of cerebrovascular event and transient ischemic attack), inpatient mortality, prolonged hospital stay (defined as length of stay greater than the median length of stay, >2 days), and increased hospitalization cost (defined as hospitalization cost >median cost of $45,949). For estimating hospitalization costs, the cost-to-charge ratios based on the Centers for Medicare and Medicaid Services reimbursement provided by the Healthcare Cost and Utilization Project were applied to the total hospital charges.^12^

### Statistical analysis

Descriptive statistics are presented as frequencies with percentages for categorical variables and as median with IQR for continuous variables. Baseline characteristics were compared using a Pearson χ^2^ test and Fisher exact test for categorical variables and the Kruskal-Wallis H test for continuous variables. For an unadjusted comparison of procedural complications and in-hospital outcomes among the study groups, the Pearson χ^2^ test was used.

For assessment of the independent association of sex with outcomes of mortality, complications, length of stay, and hospitalization costs, a single-step multivariable logistic regression model was used. Age, race/ethnicity, income, insurance status, and selected Elixhauser comorbidities (deficiency anemia, cerebrovascular disorders, congestive heart failure, chronic pulmonary disease, coronary artery disease, diabetes, hypertension, liver disease) were used for adjusted analysis. All these covariates were identified based on prior literature, bivariate analysis, and authors’ best clinical judgment. A *P* value <.05 was considered statistically significant. All statistical analyses were performed using SPSS version 26 (IBM Corp) and R version 3.6 (The R Foundation). Because of the complex survey design of the NIS, sample weights, strata, and clusters were applied to raw data to generate national estimates.^13^

## Results

A total of 1960 T-TEER procedures were performed in the US from 2018 to 2021, after applying the relevant exclusion criteria. Of these procedures, 1210 (61.7%) were performed in female patients, and 750 were performed in male patients (38.3%). Baseline characteristics of the study population are shown in Table 1. Compared to male patients, female patients undergoing T-TEER were more likely to be older. Male patients had higher burden of important comorbidities compared to female patients, including anemia (35.3% vs 27.3%, *P* < .01), coronary artery disease (58.0% vs 45.0%, *P* < .01), congestive heart failure (96.0% vs 92.1%, *P* < .01), coagulopathy (16.7% vs 10.3%, *P* < .01), liver disease (15.3% vs 7.0%, *P* < .01), and renal failure (56.7% vs 46.3%, *P* < .01). On the other hand, female patients had higher prevalence of select comorbidities compared to male patients, including atrial fibrillation (83.1% vs 80.7%, *P* < .01), hypothyroidism (28.5% vs 22.7%, *P* < .01), chronic pulmonary disease (22.7% vs 21.3%, *P* < .01), pulmonary hypertension (11.6% vs 5.3%, *P* < .01), and obesity (11.6% vs 5.3%, *P* < .01). When compared to male patients, female patients tended to have lower median income, with more patients in the lowest income quartile, (20.7% vs 15.6%, *P* < .01), and less patients in the highest income quartile (31.2% vs 36.1%, *P* < .01). All T-TEER procedures identified in this study occurred in urban hospitals, with no T-TEER procedures performed in rural hospitals.

**Table 1:** Baseline characteristics in tricuspid transcatheter edge-to-edge repair recipients, stratified by sex.

Variable	Male patients (n = 750)	Female patients (n = 1210)	*P* value
Age (mean [SD]), y	78 (71-84)	80 (72-85)	<.01
Race and ethnicity			
Caucasian	575 (79.3)	930 (77.2)	.39
African American	65 (9.0)	105 (8.7)	
Hispanic	40 (5.5)	70 (5.8)	
Other	45 (6.2)	100 (8.3)	
Comorbidities			
Anemia	265 (35.3)	330 (27.3)	<.01
Atrial fibrillation	605 (80.7)	1005 (83.1)	<.01
Congestive heart failure	720 (96.0)	1115 (92.1)	<.01
Chronic pulmonary disease	160 (21.3)	275 (22.7)	<.01
Coagulopathy	125 (16.7)	125 (10.3)	<.01
Cerebrovascular disease	30 (4.0)	55 (4.5)	.56
Diabetes	165 (22.0)	285 (23.6)	.43
Weight loss	75 (10.0)	100 (8.3)	.19
Hypertension	655 (87.3)	1070 (88.4)	.47
Hypothyroidism	170 (22.7)	345 (28.5)	<.01
Liver disease	115 (15.3)	85 (7.0)	<.01
Obesity	40 (5.3)	140 (11.6)	<.01
Peripheral vascular disorders	65 (8.7)	115 (9.5)	.53
Pulmonary hypertension	40 (5.3)	140 (11.6)	<.01
Renal failure	425 (56.7)	560 (46.3)	<.01
Coronary artery disease	435 (58.0)	545 (45.0)	<.01
Hospital location			
Rural	—	—	.02
Urban nonteaching	60 (8.0)	65 (5.4)	
Urban teaching	690 (92.0)	1145 (94.6)	
Bed size of the hospital			
Small	30 (4.0)	60 (5.0)	.23
Medium	90 (12.0)	170 (14.0)	
Large	630 (84.0)	980 (81.0)	
Census divisions			
Northeast	130 (17.3)	190 (15.7)	.46
Midwest	130 (17.3)	215 (17.8)	
South	180 (24.0)	325 (26.9)	
West	310 (41.3)	480 (39.7)	
Payee			
Medicare	620 (82.7)	1025 (84.7)	
Medicaid	10 (1.3)	25 (2.1)	<.01
Private insurance	85 (11.3)	145 (12.0)	
Self-pay	10 (1.3)	5 (0.4)	
Other	25 (3.3)	10 (0.8)	
Median income quartile			
0-25	115 (15.6)	245 (20.7)	<.01
25-50	145 (19.7)	275 (23.2)	
50-75	210 (28.6)	295 (24.9)	
75-100	265 (36.1)	370 (31.2)	

Values are n (%). For N < 11, the absolute numbers are not reported as per the Healthcare Cost and Utilization Project recommendations.

Unadjusted analysis of postprocedural complications and inpatient outcomes after T-TEER and stratified based on sex are shown in Table 2, Table 3, respectively. The prevalence of major complications was lower in female patients compared to male patients (7.4% vs 11.3%, *P* < .01). The rate of overall complications was similar between female and male patients (24.8% vs 28.0%, *P* = .12). The prevalence of pericardial effusion (2.5% vs 5.3%, *P* < .01) and cardiogenic shock (5.0% vs 10.0%, *P* < .01) was lower in female patients when compared to male patients. Female patients also experienced lower rates of acute kidney injury after T-TEER when compared to male patients (16.1% vs 28.0%, *P* < .01). Unadjusted inpatient mortality was less prevalent in female patients undergoing T-TEER compared to male patients (1.7% vs 3.3%, *P* = .02). Female patients were also noted to have a lower length of stay (2 vs 3 days, *P* < 0.01), as well as hospitalization cost ($43,631 vs $52,461, *P* < .01) when compared to male patients. Of note, non-home discharges were more prevalent in female patients than male patients (9.7% vs 5.5%, *P* < .01).

**Table 2:** Complications after tricuspid transcatheter edge-to-edge repair, stratified by sex.

Variable	Male patients (n = 750)	Female patients (n = 1210)	*P* value
Overall complications	210 (28.0)	300 (24.8)	.12
Major complications^a^	85 (11.3)	90 (7.4)	<.01
Any cardiovascular event/complication	110 (14.7)	95 (7.9)	<.01
Pericardial effusion/hemopericardium	40 (5.3)	30 (2.5)	<.01
Cardiac arrest/cardiopulmonary resuscitation	5 (0.7)	10 (0.8)	.69
NSTEMI or type II MI	25 (3.3)	25 (2.1)	.08
Heart block	20 (2.7)	20 (1.7)	.12
Cardiogenic shock	75 (10.0)	60 (5.0)	<.01
Any vascular complications	25 (3.3)	45 (3.7)	.66
Venous thromboembolism	20 (2.7)	20 (1.7)	.12
Hematoma	5 (0.7)	20 (1.7)	–
Neurological complications	10 (1.3)	5 (0.4)	–
Any bleeding or hematological complication	60 (8.0)	100 (8.3)	.07
Need for blood transfusion	45 (6.0)	90 (7.4)	.22
Any pulmonary complications	105 (14.0)	170 (14.0)	.97
Respiratory failure	50 (6.7)	105 (8.7)	.10
Long-term ventilator use	10 (1.3)	10 (0.8)	.28
Acute kidney injury	210 (28.0)	195 (16.1)	<.01

Values are n (%). For N < 11, the absolute numbers are not reported as per the Healthcare Cost and Utilization Project recommendations.

MI, myocardial infarction; NSTEMI, non-ST-elevation myocardial infarction.

a Defined as a composite of cardiovascular, vascular, hematological, and neurological complications.

**Table 3:** In-hospital outcomes and resource utilization in tricuspid transcatheter edge-to-edge repair, stratified by sex.

Variables	Male patients (n = 750)	Female patients (n = 1210)	*P* value
Died at discharge	25 (3.3)	20 (1.7)	.02
Discharge disposition			
Home discharge	685 (94.5)	1,075 (90.3)	<.01
Facility discharge	40 (5.5)	115 (9.7)	
Resource utilization, median (IQR)			
Length of stay, d	3 (1-8)	2 (1-7)	<.01
Cost of hospitalization, $	52,461.05 (37,372.79-74,115.20)	43,630.82 (32,791.77-64,900.39)	<.01

Values are n (%) unless otherwise stated.

IQR, interquartile range.

**Figure 1:**
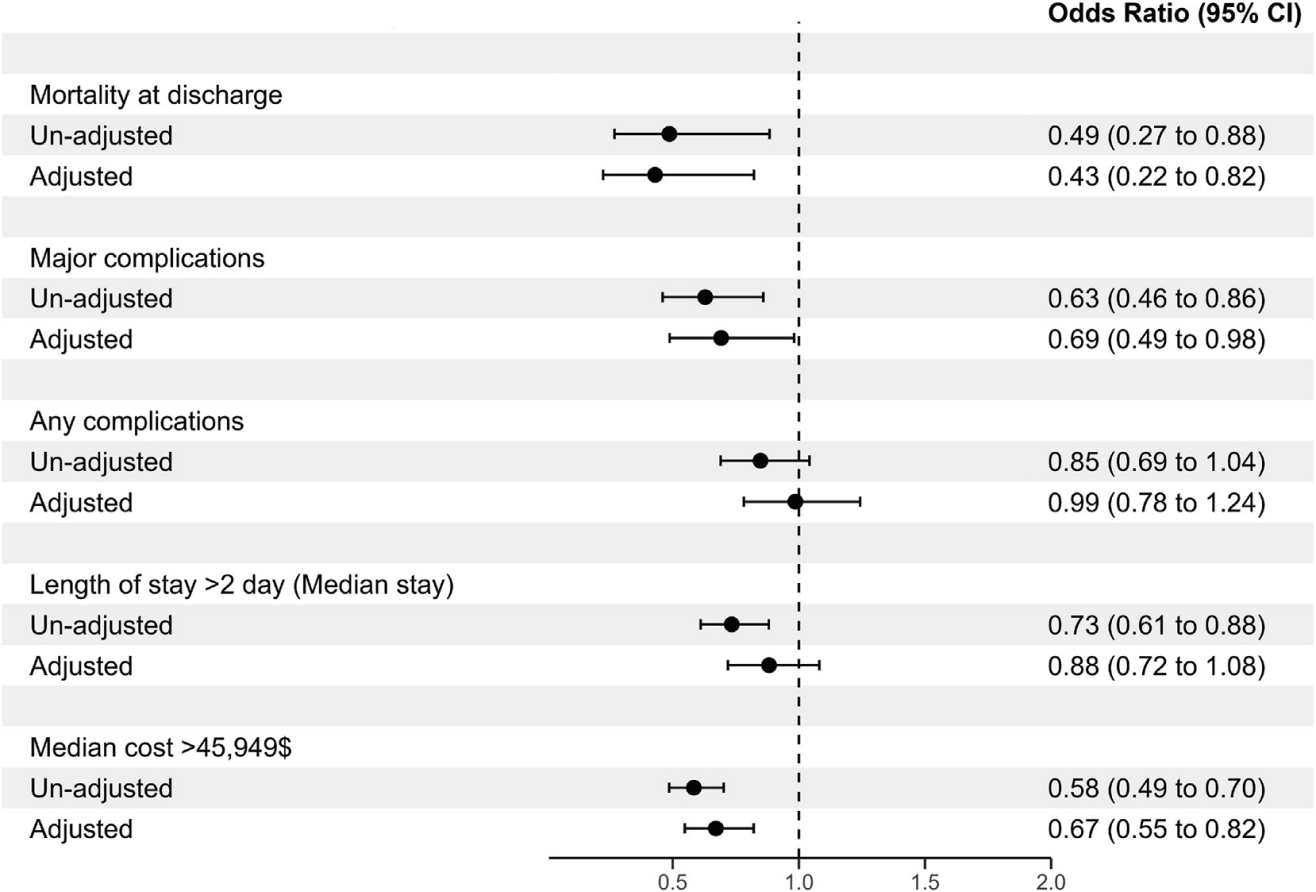
**Inpatient outcomes after tricuspid transcatheter edge-to-edge repair in female patients compared to male patients.** Forest plot depicting the independent association of sex with mortality, major complications, length of stay, and cost of hospitalization after tricuspid transcatheter edge-to-edge repair.

To assess the independent association of sex with adverse outcomes after T-TEER, multivariable models adjusting for potential confounders were created (Figure 1). After adjustment, female sex was found to be associated with lower inpatient mortality (adjusted odds ratio [aOR], 0.43; 95% CI, 0.22-0.82), lower major complications (aOR, 0.69; 95% CI, 0.49-0.98), and lower median cost of hospitalization (aOR, 0.67; 95% CI, 0.55-0.82). Overall complications (aOR, 0.99; CI, 0.78-1.24) and length of stay (aOR, 0.88; CI, 0.72-1.08) were similar after adjustment in female and male patients.

## Discussion

In this national, contemporary cohort of T-TEER procedures in patients and stratified based on sex, we report several main findings (Central Illustration) as follows: (1) female patients (61.7%) underwent more T-TEER procedures in the US than male patients (38.3%); (2) female patients undergoing T-TEER were more likely to be older and experienced lower prevalence of important comorbidities compared to male patients, with a few notable exceptions; and (3) female sex was independently associated with lower mortality, major complications, and hospitalization costs.

**Central Illustration:**
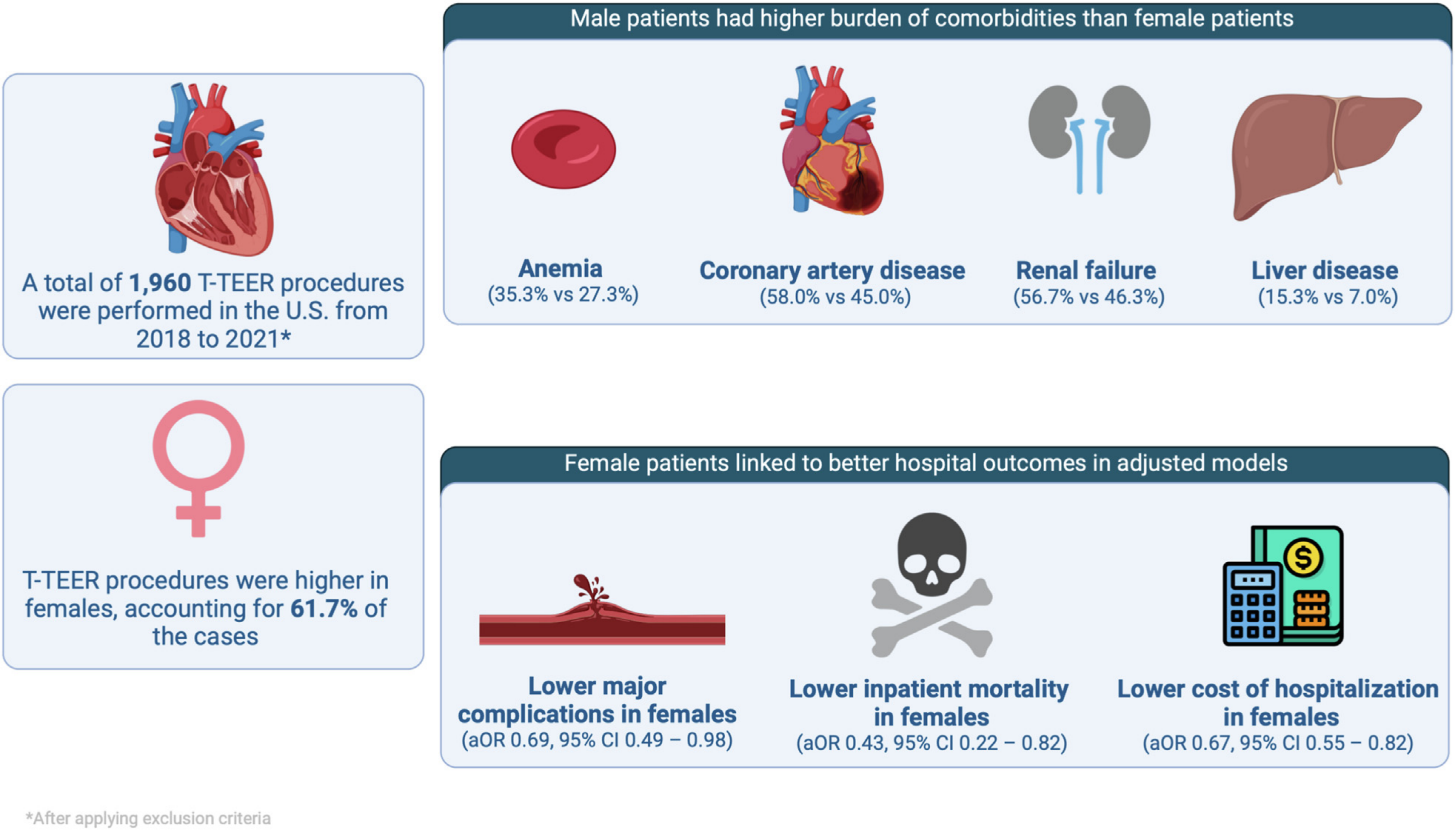
**Major comorbidities and outcomes of tricuspid transcatheter edge-to-edge repair (T-TEER) in female patients compared to male patients.** T-TEER procedures were more prevalent in female patients than male patients. Male patients had higher rates of important comorbidities than female patients. After adjusting for confounders, female patients had better hospital outcomes in terms of major complications, mortality, and cost of hospitalization. aOR, adjusted odds ratio.

Tricuspid regurgitation has been strongly associated with increased cardiovascular morbidity and mortality, partly due to the limited medical therapies available to treat it.^2^^,^^3^ Surgical repair or replacement for TR has been shown to have operative mortality rates of up to 4%.^14^ Thus, cardiac surgery for isolated TR is seldom performed.^8^ Consequently, many patients with severe TR who are deemed too high a surgical risk are often left without an interventional option. This high surgical risk has been associated with underlying comorbid conditions, as well as complications from TR itself, including right heart failure, pulmonary hypertension, and venous congestion.^1^ Thus, a minimally invasive approach to TR repair was essential. The TriValve and TRILUMINATE studies have demonstrated high success rates of T-TEER implantation, with corresponding improvements in severity of TR and quality of life when compared to medical therapy alone, all of which were sustained in long-term follow-up.^1^^,^^3^^,^^4^^,^^7^ Furthermore, when compared to surgical repair, transcatheter repair has been shown to have improved safety and short-term mortality.^15^, ^16^, ^17^ With the advent and advancement of transcatheter valvular repairs, disparity outcomes have also been investigated, such as sex-specific outcomes. Female patients undergoing mitral TEER have been shown to have worse procedural outcomes, and similar inpatient mortality rates, but lower short- and long-term mortality than male patients.^8^, ^9^, ^10^, ^11^^,^^18^ On the contrary, in recent single-center and multicenter studies, patients undergoing T-TEER have been shown to have similar procedural complications, inpatient mortality rates, short- and long-term major adverse cardiovascular events, and mortality, regardless of sex.^2^^,^^8^^,^^19^^,^^20^ Our study is the first large national study to demonstrate significantly lower inpatient mortality rates in female patients undergoing T-TEER. Notably, female patients were also noted to have significantly lower rates of major and cardiovascular complications, and similar rates of other adverse outcomes. In particular, male patients in our study had higher rates of pericardial effusion and cardiogenic shock. Prior investigations of in-hospital outcomes after transcatheter mitral valve repair have shown no sex-related differences in rates of pericardial effusion, cardiac tamponade, and cardiogenic shock.^18^^,^^21^ Other studies regarding transcatheter aortic valve replacement found that female patients had higher rates of pericardial complications, including tamponade, hemopericardium, and effusion requiring pericardiocentesis.^22^^,^^23^ It is unclear why male patients in our study were more predisposed to pericardial effusion after T-TEER. The etiology of pericardial effusion has been postulated to be multifactorial, including intraprocedural perforation, cardiac vessel rupture, ongoing inflammation, and postcardiac injury syndrome.^23^^,^^24^ Further research into sex-specific risk factors is encouraged to help reduce sex-based disparities, given presence of pericardial effusion after transcatheter valvular repair has been associated with increased cost of stay and in-hospital mortality.^23^^,^^24^

As consistent with prior literature on transcatheter valve repair, our study also found that male patients generally had higher rates of comorbid conditions than female patients, with a few important exceptions.^5^^,^^9^, ^10^, ^11^ In particular, female patients in our study had higher rates of atrial fibrillation, hypothyroidism, obesity, chronic pulmonary disease, and pulmonary hypertension. This is in contrast to prior studies, in which rates of atrial fibrillation and chronic obstructive pulmonary disease were similar in patients undergoing T-TEER, regardless of sex.^2^^,^^5^^,^^8^^,^^20^ Prior literature has suggested that atrial fibrillation is a clinical predictor for the progression of TR, with a higher risk in female patients due to hypothesized sex-related differences in tricuspid valve annular structure.^3^, ^4^, ^5^ Rates and severity of pulmonary hypertension have also been noted to be higher in female patients with TR.^5^^,^^19^ The progression of pulmonary hypertension can contribute to the worsening of TR, and vice versa.^5^ The increased rates of atrial fibrillation and pulmonary hypertension in female patients in our study may be contributing to the greater prevalence of TR in female patients, and thus the greater majority of female patients undergoing T-TEER. Further research regarding risk factors and anatomical differences in the incidence and severity of TR among sexes would be insightful.

In terms of resource utilization, female patients in our study were noted to have significantly lower cost of hospital stay after tricuspid repair, as has been noted in current literature.^19^ There were no significant sex-based differences in length of stay, which is consistent with prior studies for transcutaneous mitral or tricuspid valve repair.^8^^,^^10^ Overall, similar or better outcomes for resource utilization and inpatient mortality in female patients are likely related to the lower rates of comorbid conditions and inpatient complications.

### Limitations

The results of our current study should be interpreted in the context of the following key limitations. First, the NIS relies on administrative coding for disease and procedure identification which may be subject to errors. However, it should be noted that the NIS uses a rigorous data quality control program to minimize miscoding and ensure the integrity of data. Second, as the NIS includes data on inpatient stays only, long-term outcomes and mortality cannot be ascertained. Furthermore, no data on changes in quality of life or functional capacity are available from the NIS. Third, the NIS database does not differentiate between primary and secondary TR, nor does it specify the underlying etiology of TR, which may have important implications on procedural outcomes. Fourth, the NIS database does not provide data on procedural success, such as improvement in TR grading. Fifth, there are no data available on procedural factors of T-TEER, such as utilization of contrast, length of procedure, operator experience, or volume of device placement per hospital.

## Conclusion

In conclusion, our large, contemporary study of real-world T-TEER procedures in the US depicted important sex-based disparities regarding procedural outcomes and resource utilization. After multivariate adjustment, female sex was associated with lower in-hospital mortality and better or similar outcomes and resource utilization than male sex. Further investigation into potential etiologies driving these disparities is encouraged in order to identify and address any intervenable sex-based disparities. Future large-scale investigations on the long-term outcomes and success of T-TEER stratified by sex would also be insightful.
