# Supplementary material for: Inpatient Outcomes of Tricuspid Transcatheter Edge-to-Edge Repair in the United States Based on Sex
Source: J Soc Cardiovasc Angiogr Interv. 2025 May 1;4(6):102644. doi: 10.1016/j.jscai.2025.102644 (PMC12230491; doi:10.1016/j.jscai.2025.102644)
Supplement: Supplementary Material [file mmc1.docx]

# ICD 10 codes

**Tricuspid Percutaneous Repair Device:**

02UJ3JZ

**Complications definition:**

1. Major complications: Shock, CPR, NSTEMI, STEMI, pericardiocentesis, arterial embolism, GI bleed, vascular complications.
2. Cardiac complications: CPR/resuscitation, heart block, Myocardial infarction, Pericardiocentesis, cardiogenic shock, tamponade.
3. Systemic complications: septic shock, arterial embolism, anaphylaxis.
4. Vascular complications: venous thrombosis, hemoperitoneum, hematoma, pseudoaneurysm, arteriovenous fistula.
5. Neurological complications: TIA, ischemic stroke, hemorrhagic stroke.
6. Gastroenterological/hematological complications: GI bleed, bleeding during procedure, bleeding requiring transfusion.
7. Pulmonary complications: pneumothorax, bacterial pneumonia, prolonged ventilator use, acute respiratory failure.
8. **Cardiovascular:**
9. Cardiac Arrest/CPR procedure code: 5A12012
10. STEMI: I2101, 02, 03, 09, i2111, i219, i212, i2121
11. NSTEMI or type II MI: I21A,I21A1, I21A9, I214
12. Air Embolism: T800XXA
13. Heart Failure: I5021, 23, 31, 33, 41, 43
14. Heart Block, complete: I442
15. Percutaneous coronary intervention: 0270, 0271, 0272, 0273
16. Pericardial effusion/Hemopericardium: I31.2

10. Cardiac Tamponade: I314

11. Pericarditis: I300, I301, I308, I309

12. Need for Pericardiocentesis: 0W9D3, 0W9D4

13. Cardiogenic Shock: R570, T8111XA

14. Need for diagnostic left heart catheterization: B2100, B2101, B211, B212, B213, B215

**B. Systemic:**

15. Anaphylaxis: T78

16. Arterial thrombosis: I74x

17. Deep venous thrombosis: i82

18. Septic shock: R65.21

**C. Vascular complications:**

19. AV fistula: I300, I308, I309

20. Pseudoaneurysm: I72

21. Local site hematoma: M7981, L763

22. Local site bleeding: L760, L761, L762

23. Retroperitoneal Bleeding: K661

23. Dissection: I77.7, I71.0

**D. Neurologic:**

24. Hemorrhagic stroke: I60, I61, I62

25. Ischemic stroke: I63

26. TIA: G45

**E. Hematological complications:**

27. GI bleeding: K92

28. Hemothorax after procedure or unknown: J942

29. Need for blood products transfusion: 3023x

**F. Pulmonary:**

30. Post procedure or iatrogenic pneumothorax and air leak: J93.5

31. Pleural Effusion: J90, J918

32. Pneumonia bacterial: J13-J18

33. Pulmonary embolism: I26

34. Respiratory failure: J960, J962, J969

**G. Kidney Injury**

35. Hemodialysis procedure code: 5A1D70Z, 5A1D80Z, 5A1D90Z, 5A1D00Z, 5A1D60Z

36. AKI: N17
